# Neuraxial anaesthesia in the parturient with pre-existing structural spinal pathology

**DOI:** 10.1016/j.bjae.2024.05.005

**Published:** 2024-08-01

**Authors:** G. Crowe, T. Drew

**Affiliations:** 1 The Rotunda Hospital, Dublin, Ireland; 2 Beaumont Hospital, Dublin, Ireland; 3 RSCI University of Medicine and Health Sciences, Dublin, Ireland

**Keywords:** epidural anaesthesia, obstetric anaesthesia, spinal anaesthesia, spinal diseases

**Key points**

• Structural pathology of the lower spine is common in pregnant women, but rarely represents a contraindication to neuraxial anaesthesia.
• Antepartum review by the anaesthetist is essential to determine if a neuraxial technique is possible, what difficulties may be encountered, and the likely efficacy of a neuraxial technique.
• Women who have undergone spinal surgery may have abnormal anatomical landmarks and an abnormal or absent ligamentum flavum (precluding a loss-of-resistance technique).
• Disruption of the epidural space after spinal surgery can limit the spread of local anaesthetic drugs.

**Learning objectives**

By reading this article, you should be able to:

• Explain the basic anatomy of the lumbar vertebral column as applied to neuraxial anaesthesia.
• Describe commonly encountered structural spinal pathologies in the parturient and their potential implications for neuraxial anaesthesia.
• Discuss approaches to neuraxial anaesthesia in the parturient with abnormal spinal anatomy.

Pathology of the lower back and vertebral column is common in pregnant women and represents approximately 17% of referrals to obstetric anaesthesia preassessment clinic.^1^ Common issues include simple mechanical low back pain, a history of spinal surgery, congenital spinal dysraphisms (including spina bifida, meningocoele and myelomeningocoele), lumbosacral neuropathies and scoliosis. The patient's main concerns may include whether they are suitable for neuraxial anaesthesia, how well it will work and if it will worsen their existing condition. These questions present a challenge for the anaesthetist as there is a diverse range of pathologies each with their own distinct implications for neuraxial anaesthesia. Furthermore, there is limited guidance or consensus in the literature. This can disrupt the process of informed consent and may impact clinical decision making, excluding some women from neuraxial anaesthesia out of an excess of caution. The aim of this article is to provide an overview of commonly encountered structural disorders of the vertebral column and their impacts on the feasibility and risks of neuraxial anaesthesia (Table 1).

**Table 1:** Summary of clinical features, potential risks and possible techniques for neuraxial anaesthesia and analgesia in the parturient with abnormal vertebral or spinal anatomy.

Pathology and clinical features	Risks/Patient counselling	Techniques
**Mechanical low back pain** • Dull lumbar region pain • Exacerbated by forward flexion, erector spinae palpation • Lumbar movement may be limited • May radiate into leg, but usually not below the knee	• Neuraxial anaesthesia will not increase risk of postpartum back pain^2^ • Patients who have pre-existing back pain are at increased risk of having postpartum back pain^3^	• Epidural and spinal anaesthesia/analgesia can be used
**Herniated lumbar disc/spinal stenosis** • Back pain and unilateral leg pain that radiates below the knee • Numbness, paraesthesia, weakness and/or loss of tendon reflexes in nerve root distribution. • Pain on straight leg raise • Pain is relieved by lying down and exacerbated by prolonged walking/sitting	• Extremely rare reports of worsening neurological impairment in obstetric patients with existing spinal stenosis or disc herniation when neuraxial is used^4^, ^5^, ^6^, ^7^, ^8^	• Epidural and spinal anaesthesia/analgesia can be used • Currently no compelling evidence to avoid neuraxial anaesthesia at the level of disc herniation in asymptomatic disc disease • If known severe disease, or symptoms suggestive thereof, consider risk–benefit of neuraxial • Consider avoiding affected level, and have increased vigilance for postoperative neurological impairment^9^ • Ultrasound may be helpful for localising unaffected levels
**Spinal stenosis** • Low back, buttock or leg pain exacerbated by standing, walking, lumbar extension. Relieved by forward flexion, sitting, lying flat. May be burning or cramping in nature • Gradual onset pain, numbness and weakness after walking a predictable distance. Less pain walking uphill.		
**Spondylolysis and spondylolisthesis** • Low back pain, exacerbated by extension	• No contraindication or increased risk with neuraxial in spondylolysis • There may be a slightly increased risk of dural puncture in spondylolisthesis if epidural is attempted at the level of pathology	• Epidural and spinal anaesthesia/analgesia can be used • Consider avoiding affected level in spondylolisthesis. • Ultrasound may be useful for localising unaffected levels
**Uncorrected scoliosis** • History of diagnosed scoliosis • Physical evidence of spinal curvature on exam	• Success rates for epidural and spinal anaesthesia in uncorrected scoliosis are almost comparable to those without spinal deformity, but the procedure may take longer and may require more attempts^10^, ^11^, ^12^ • Unilateral block is more common, but is usually manageable with positioning/additional local anaesthetic	• Epidural and spinal anaesthesia/analgesia can be used • Consider a paramedian approach on the convex side of the curve, or needle direction towards the convex side from the apparent midline • Positioning and higher volumes of local anaesthetic may be required to overcome unilateral block
**Corrected scoliosis** • History of scoliosis with corrective surgery	*Anterior fusion:* • There should be no increased risk of complications or difficulty with neuraxial anaesthesia/analgesia *Posterior fusion:* • Neuraxial anaesthesia may not be possible in certain patients (lumbar/lumbosacral fusions) • In those in whom neuraxial anaesthesia is possible, scar tissue and anatomical distortion may make the procedure more difficult. Both epidural and spinal may take longer and may require more attempts • Scar tissue may also result in unpredictable analgesia. Patchy or unilateral analgesia can often be overcome with positioning and/or additional local anaesthetic • Ultimately, analgesia is usually achievable, although rescue techniques (e.g. spinal anaesthesia) may be required^13^	• Epidural and spinal anaesthesia/analgesia can be used in unfused segments • Imaging and medical notes should be reviewed to ascertain which level may be suitable • Ultrasound may be helpful for localising suitable levels • Positioning and higher volumes of local anaesthetic may be required to overcome unilateral block • Spinal anaesthesia can be used as a rescue technique where epidural has failed
**Previous spinal surgery** • History	• No contraindication to neuraxial technique • Scar tissue from previous surgery may make analgesia unpredictable • Depending on nature of surgery, if neuraxial is attempted at the operative level, there may be an increased risk of dural puncture	• Epidural and spinal anaesthesia/analgesia can be used safely in unoperated segments • Because of variability in ligamentum flavum resection in various surgeries, it is prudent to avoid insertion at operative level • Ultrasound may be useful to localise appropriate level
**Spinal dysraphism** • History of operative repair • History of close spinal dysraphism, may have neurological impairment	*Previous repair* • There is a risk of damage to the spinal cord if epidural or spinal anaesthesia is performed at the level of a previous repair or where there is spinal cord tethering • Epidural anaesthesia may be possible above the level of repair, although it may be suboptimal owing to scar tissue and abnormality of the epidural space • Spinal anaesthesia has been successfully used above the level of the repair *Closed isolated bony defects* • No contraindication to neuraxial anaesthesia • Theoretical increased risk of dural puncture	• Epidural and spinal techniques can be used in some patients • If used, imaging/medical notes should be carefully reviewed to ascertain at which level such techniques are safe (e.g. where there is no spinal cord tethering and the epidural anatomy is normal) • Ultrasound may be useful to localise the suitable levels at the bedside • Epidural anaesthesia, if used, may be unpredictable as a result of abnormal epidural anatomy • Low-dose spinal anaesthesia at low thoracic levels can be considered

## Applied anatomy of the vertebral canal

The management of labour pain using neuraxial analgesia requires an effective neurological block spanning from T10/L1 to S2–4; this may be achieved through the insertion of an epidural catheter or a combined spinal epidural (CSE) technique. Conversion of labour analgesia to anaesthesia for Caesarean delivery requires extension of the block to the level of T4.

**Fig 1:**
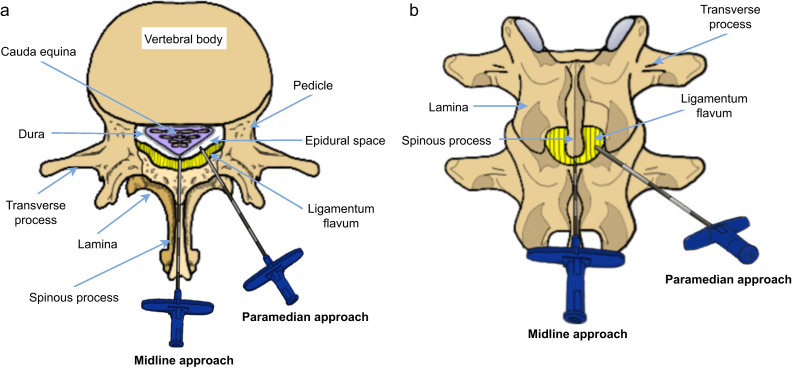
(a) Cross-sectional view of the lumbar region, with needle position for midline and paramedian approaches to the neuraxis. (b) Posterior view of lumbar vertebra including ligamentum flavum, with needle position for midline and paramedian approaches to the neuraxis.

The vertebrae in the lumbar region have an anterior body and posterior arch composed of posterolateral pedicles, transverse processes and posterior laminae that fuse centrally to form spinous processes. Within the posterior arch lies the spinal canal. Vertebral foramina may be found laterally, bordered by the pedicles, transverse processes and bodies of the adjacent vertebrae (Fig. 1).

Inside the spinal canal lies the epidural space, which is a potential space approximately 5–6 mm in width and anteroposterior diameter at lumbar levels. The space contains the dural sac, spinal nerves, vessels, connective tissues and fat. The spinal cord is contained within the dural sac along with the pia and arachnoid membranes that surround the cord and separate the epidural, subdural and subarachnoid spaces. The spinal cord terminates as the conus medullaris in the lumbar region, most commonly at the lower border of L1, but in some cases may terminate as low as L3. Below this level, the cauda equina lies free within the subarachnoid space as the spinal nerves within continue towards their respective foramina.

Several important ligaments that stabilise the vertebral column posteriorly are encountered during epidural or spinal needle insertion. The supraspinous ligament connects the tips of the spinous processes from C7 to the sacrum. Interspinous ligaments lie deep to this, connecting adjacent spinous process to one another. Fused to the inner surface of the supraspinous ligament is the ligamentum flavum, a dense structure high in elastin that connects adjacent vertebral laminae. In the lumbar region, the ligamentum flavum is approximately 3–5 mm thick. The ligamentum flavum forms as two halves, and midline fusion may be deficient in 10–20% of women in the lumbar region.^14^

The most common practice used for lumbar epidural insertion in labour is the loss-of-resistance technique at L3/L4 or L4/L5. The epidural space is localised by the sudden, discrete loss of resistance to injection encountered as the needle passes through the dense ligamentum flavum into this low-pressure space. A midline or paramedian approach may be used; in both, the ligamentum flavum yields an increase in resistance to needle advancement, which heralds the imminent arrival in the epidural space (Fig. 1).

When assessing the feasibility of and approach to neuraxial analgesia in a patient with abnormal spinal anatomy, several key factors should be considered:

(i) Are anatomical landmarks abnormal or missing, and does a specific vertebral level need to be avoided?
(ii) Does the patient have an intact ligamentum flavum and will a loss-of-resistance technique be feasible?
(iii) Is there tissue, such as scar tissue, bone graft or surgical spinal implants in the path of the needle that could preclude needle insertion or be damaged by needle or catheter insertion?
(iv) Is there significant disruption to the epidural space that would preclude local anaesthesia spread or catheter insertion?

## Mechanical low back pain

Low back pain in pregnancy affects up to 75% of pregnant women, with typical onset after 22 weeks' gestation.^15^ The aetiology of pregnancy-associated low-back pain is multifactorial. Increased joint and ligamentous laxity, anterior pelvic tilt and compensatory lumbar hyperlordosis often contribute.^16^

There is no contraindication to neuraxial analgesia in pregnant women with simple mechanical low back pain. A Cochrane review including more than 1806 patients demonstrated that epidural analgesia had no statistically significant impact on the risk of long-term backache.^2^ Thus, women may be reassured that an epidural will not increase the risk of long-term back pain. Those women with pre-existing back pain before pregnancy are at an increased risk of developing postpartum back pain irrespective of neuraxial anaesthesia.^3^ Low back pain may accompany radicular pain with associated motor and sensory deficits, which should be examined and clearly documented. Back pain accompanied by red flag symptoms such as saddle anaesthesia, major motor weakness, incontinence and fevers should prompt further investigation.

## Disc disease and spinal stenosis in pregnancy

Lumbar spinal stenosis is characterised by narrowing of the lumbar spinal canal, the neural foramina or both, with compression of the spinal nerve roots. A common cause of spinal or foraminal stenosis in younger people is lumbar disc protrusion, although osteophyte formation, facet joint hypertrophy and ligamentum flavum buckling and hypertrophy may also contribute. Lumbar disc disease is common amongst women of childbearing age. Up to 30–50% of asymptomatic individuals between 20 and 40 yrs of age will have radiological evidence of lumbar disc herniation or bulging.^17^ Symptomatic lumbar disc herniation is less common and affects approximately 1–3% of the population per year. Anatomical and hormonal changes during pregnancy may predispose to increased lumbar disc instability; however, lumbar disc herniation amongst pregnant women is less common than in the general population, with data from the 1980s suggesting an incidence of one in 10,000.^18^

In recent years, some evidence has emerged to suggest that there may be an increased risk of new or worsening neurological impairment in patients with existing spinal stenosis when neuraxial anaesthesia is used.^4^, ^5^, ^6^, ^7^ This may be related to a compressive–ischaemic effect because of reduced cross-sectional area of the vertebral canal, direct trauma, haematoma, or enhanced neurotoxic effects of local anaesthetics resulting from limited distribution in the smaller space. It is important to note that these data are almost entirely derived from older, non-obstetric patients, with very few cases reported in parturients.^8^ Furthermore, although there is an association between spinal stenosis and injury after neuraxial block, often the diagnosis of spinal stenosis was made during workup for the injury. There is no clear evidence that spinal stenosis led to these injuries. As noted above, a significant proportion of patients with some degree of spinal stenosis secondary to disc herniation will be asymptomatic, and in these cases the vast majority will have neuraxial anaesthesia without issue.

In women with known severe spinal stenosis or symptoms suggestive thereof, societal guidelines recommend that the risk–benefit relationship be considered, and if neuraxial block is performed, there should be increased perioperative vigilance for symptoms suggestive of neural compromise.^9^ Fortunately, there is no firm linkage to injury if spinal stenosis is at a site distant from the level of neuraxial block placement, and if possible, the neuraxial block can be performed at an alternative level to the one affected by disc herniation or stenosis.

## Spondylolisthesis

Spondylolysis is an anatomical defect in the vertebral pars interarticularis, which joins the upper and lower articular processes. It most commonly occurs in the low lumbar vertebrae (L5 more commonly than L4), typically in active adolescents. Spondylolisthesis refers to displacement of a vertebral body on the one below caused by intersegmental instability of the vertebrae, creating either anterior (anterolisthesis) or posterior (retrolisthesis) translation of a vertebra in relation to the segment below. The two major causes of spondylolisthesis are isthmic (associated with spondylolysis) and degenerative (associated with degenerative arthritis of the posterior facet joints, intervertebral discs or both).

Degenerative spondylolisthesis is more common in women. Pregnancy may be a major independent factor for the development of the condition, although women with a previous diagnosis of spondylolisthesis have not been shown to have an increase in vertebral body translation during pregnancy.^19^

The prevalence of both spondylolysis and spondylolisthesis in the general population is approximately 6–11% and may be higher in more active individuals.^20^ Many patients with spondylolysis and spondylolisthesis are asymptomatic, but spondylolysis is the most common radiologically identifiable pathology within adolescents complaining of low back pain,^21^ and thus some women may have already had imaging and a diagnosis before pregnancy.

**Fig 2:**
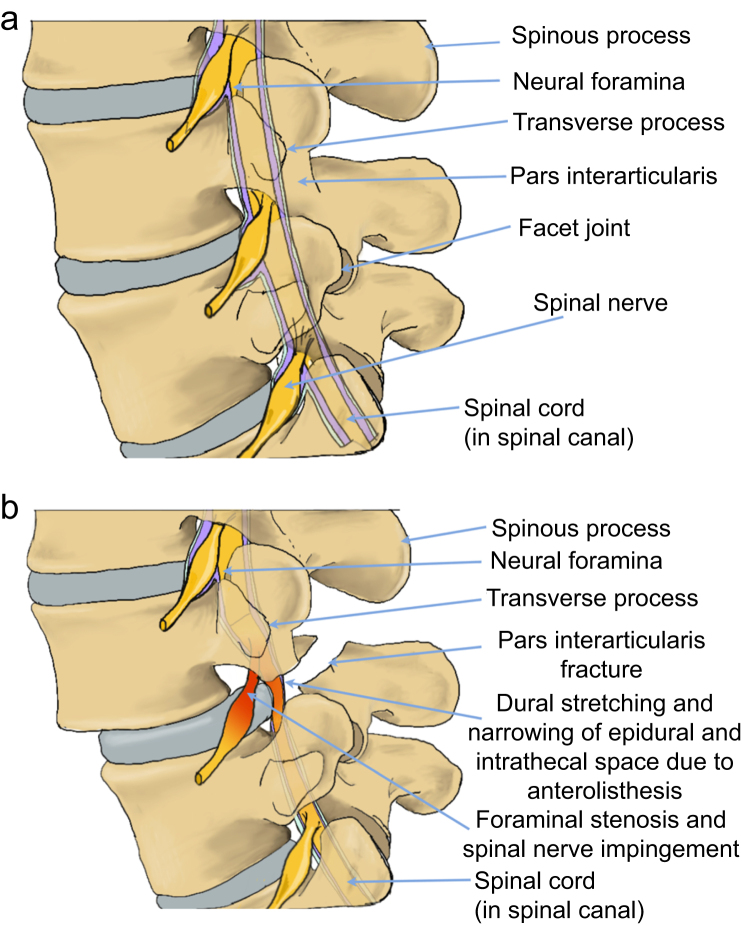
(a) Normal bony anatomy of the lumbar vertebrae. (b) Lumbar spondylolisthesis with anterior translocation of vertebra on the one below (anterolisthesis) with resultant foraminal stenosis and spinal nerve impingement and stretching of the dura at the affected level.

Spondylolysis is usually asymptomatic but may present with low back pain that is worse with extension. Spondylolisthesis may also present with back pain, and symptoms of a radiculopathy caused by compression of the nerve roots in the lateral recess or in the foramen (Fig. 2).

Isthmic spondylolisthesis and degenerative spondylolisthesis are most commonly found at L5/S1 and L4/L5 vertebral levels, respectively, areas commonly chosen for neuraxial anaesthesia procedures.^22^ Neither condition presents a contraindication to neuraxial anaesthesia; however, the translocation of one vertebra over the other can cause stretching of the dura and contraction of the epidural space, possibly increasing the risk of inadvertent dural puncture (Fig. 2). It is prudent to avoid this lumbar level to reduce this risk. In women with a diagnosis of spondylolysis, there is no contraindication to neuraxial anaesthesia at the affected spinal level.

## Scoliosis

Scoliosis is defined as a ≥10° lateral curvature of the spine in the standing position on a coronal radiographic image; the incidence is 2% in the general population.^23^ Scoliosis may arise from neuromuscular disease or congenital anatomical anomalies; however, idiopathic scoliosis is the most common aetiology with adolescent idiopathic scoliosis (AIS) encompassing 80% of cases. AIS disproportionately affects females, who are also more likely to have severe curves and thus require surgical correction.^23^

Management of scoliosis depends on skeletal maturity, magnitude of deformity (as measured by Cobb angle) and curve progression. Operative management is indicated when progressive scoliosis exceeds 45° in patients with an immature skeleton or when progression or pain occurs after skeletal maturity. These patients may undergo a series of procedures followed by definitive fusion in adolescence.^23^ Posterior fusion is most frequently performed, although anterior or combined posterior–anterior approaches may be indicated in some patients.

Neuraxial anaesthesia in women with both corrected and uncorrected scoliosis is possible, but it may be more technically challenging to perform and may provide suboptimal analgesia compared with those without scoliosis.

### Uncorrected scoliosis

**Fig 3:**
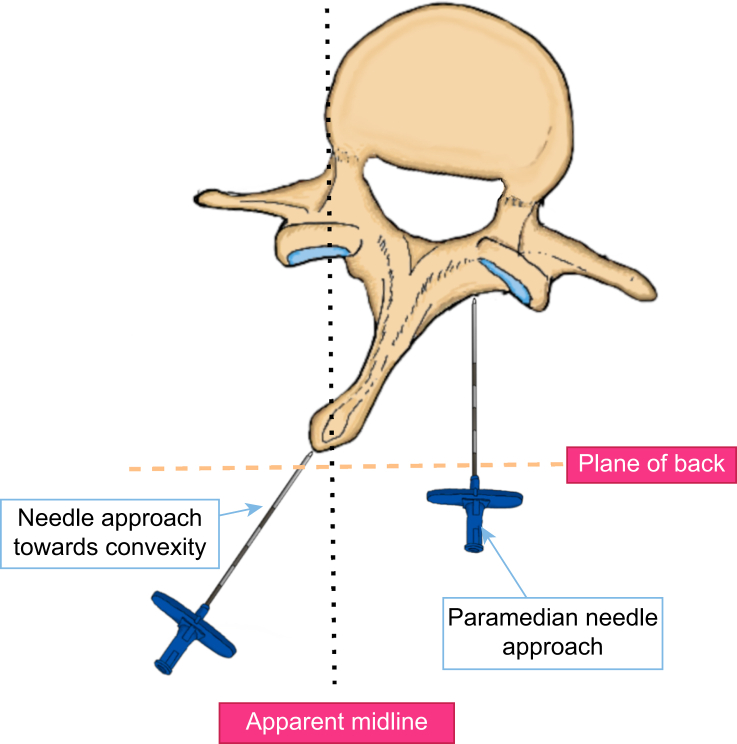
Paramedian *vs* oblique approaches to the neuraxis in scoliosis. An apparent midline approach may result in a needle trajectory that is, in reality, significantly off-midline.

**Fig 4:**
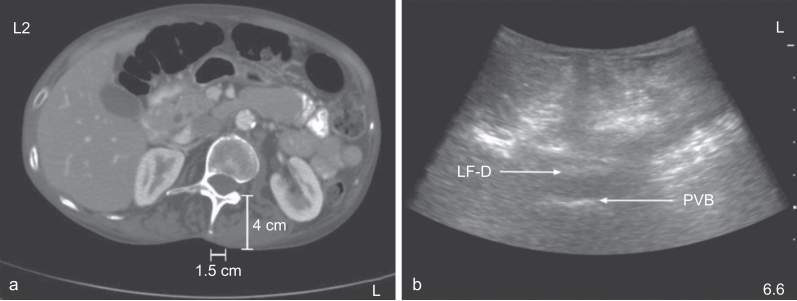
(a) Axial CT demonstrating L2 vertebral body rotation and pathway for paramedian approach to the epidural space. (b) Transverse ultrasound image depicting anatomy consistent with the CT. LF-D, ligamentum flavum–dura mater complex; PVB, posterior vertebral body.

In uncorrected scoliosis, rotation of the spine can distort surface anatomy, resulting in deviation of the midline of the epidural space towards the convexity of the curve. Thus, the needle should be directed toward the convexity of the curve in these cases. Alternatively, using a paramedian approach, a direct path to the neuraxial spaces can be found on the convex side of the curve (Fig 3, Fig 4).^24^ Ultrasound is helpful in locating the true midline and identifying the appropriate intervertebral level for intervention.^25^ The epidural injectate has been shown to initially flow towards the concave side of the curve in approximately 80% of cases. As such analgesia may be unilateral and higher volumes may be required.^26^

The published literature pertaining to success rates for epidural and intrathecal analgesia in uncorrected scoliosis is limited. A 2009 literature review including 24 procedures revealed success rates of 80% and 73% for epidural and intrathecal anaesthesia, respectively, with difficult or multiple attempts at placement in 4%.^10^ More recent data, again only including small numbers, suggest better success rates close to 100% for both procedures.^11^^,^^12^

### Corrected scoliosis

In anterior fusion, the vertebral column is accessed anteriorly with screw and rod placement lateral to the vertebral bodies. The spinous processes remain intact and no bone graft or scar tissue should be present posteriorly to inhibit needle placement. Neuraxial analgesia in these patients should be straightforward.

Posterior fusion entails the placement of multiple bilateral pedicle screws, hooks or both, followed by rod insertion and compression/distraction of the convex and concave aspects of the curve. Spinal osteotomies may be required for release before compression/distraction, and the procedure is completed with placement of extensive bone graft to encourage fusion of the involved segments. As a result, access to the neuroaxis in the region of the fusion may be impossible. Furthermore, distraction/compression, bone grafting and spinal osteotomies may cause epidural space distortion and fibrosis, which may affect catheter passage and spread of local anaesthetic. Up 30% of patients undergoing surgery will require some degree of lumbar fusion, which may severely limit accessible interspaces.^27^ Fusion surgery should not involve the sacrum to preserve lumbosacral mobility. Incision scars will often extend slightly below the level of the fusion and as such do not necessarily correlate with the exact fusion level.

Epidural catheterisation may be challenging in women who have had posterior fusion, with catheter placement taking longer and failed or repeated attempts being more common than in healthy controls. Reassuringly, despite these difficulties, successful analgesia in this group has been reported in up to 88% of cases.^13^ The reported rate of successful spinal anaesthesia in the literature is approximately 70%,^10^ but this is likely an underestimate. More recent data from patients with mixed corrected and uncorrected scoliosis suggest higher success rates, although exact figures for subarachnoid blocks in patients with corrected scoliosis specifically are not available.^11^^,^^28^ Notably, spinal analgesia has been used as a successful rescue technique in failed epidural catheterisation in this group.^12^^,^^13^

Parturients with scoliosis should be identified early and preassessed before labour to facilitate counselling regarding the potential challenges and complications, and to institute contingency plans in the event that neuraxial analgesia is not possible. Ideally surgical documentation and radiological investigations should be available to assess the level and extent of vertebral involvement. In corrected scoliosis, neuraxial analgesia below the level of instrumentation is an option and the use of ultrasound for vertebral level localisation is recommended. Common inadequacies in analgesia such as unilateral or patchy block may be overcome through repositioning and additional doses of local anaesthetic as required. In the event of epidural failure, primary or repeat spinal anaesthesia may be a rescue option with or without the use of a spinal catheter.^13^^,^^29^

## Previous spinal surgery

Lumbar spinal surgery is becoming increasingly frequent, with approximately 25% of all surgeries undertaken in patients aged <40 years.^30^ Common procedures performed in this age group include discectomy, laminectomy and lumbar fusion, which may be conducted via a minimally invasive approach. Scar tissue-mediated distortion of anatomical landmarks and epidural fibrosis may be an issue for neuraxial anaesthesia in these patients. Furthermore, if the ligamentum flavum has been resected, a loss-of-resistance technique at the surgical level will not be reliable. Discectomy, either open or minimally invasive, may entail some degree of laminotomy and resection of the ligamentum flavum to allow access the disc, although this may not be required in lateral or transforaminal approaches. Decompressive laminectomy or laminotomy surgery also frequently includes a ligamentum flavum resection. Interbody fusion procedures often also require some posterior decompression and include a ligamentum flavum and laminar resection, followed by pedicle screw insertion.

As in corrected scoliosis, surgical and radiological documentation should be sought to enable targeting of an interspace distinct from the surgical site, if feasible, to assist successful catheter insertion and anaesthetic spread.

## Spinal dysraphism

Spinal dysraphism encompasses a broad spectrum of congenital anatomical abnormalities of the vertebral arches, spinal cord and meningeal structures. They are among the most common birth defects, with an incidence of approximately 0.3/1000 births.^31^ Spinal dysraphisms are broadly divided into open and closed abnormalities. Open spinal dysraphisms are characterised by exposure of the nervous tissue, the meninges or both to the environment through a congenital bony defect. Closed spinal dysraphisms are covered by skin such that there is no exposure of the neural/meningeal tissues, although there may be overlying cutaneous stigmata such as skin dimples or hypertrichosis. The underlying spinal cord and associated structures are abnormal; there may be low-lying or tethered spinal cord and conus medullaris, split cord or lipomata. More than 80% of spinal dysraphisms, either open or closed, occur in the lumbosacral region.^32^ Isolated bony defects in the vertebral arch are encompassed within the spectrum of closed dysraphisms; however, their clinical significance in asymptomatic patients is disputed and have been considered as a normal variant.^33^

The wide range of presentations and clinical manifestations of spinal dysraphisms represent a challenge to the obstetric anaesthetist. There are limited case reports and case series in the literature describing how to manage these women.

Sensory impairment is extremely variable and may be asymmetric or demonstrate perineal sparing. Coexistent scoliosis and Chiari malformation with hydrocephalus may be present.^34^ Such patients also may have severe latex allergy.^35^ Patients with open spinal dysraphism will usually have undergone surgical closure in infancy; the spinal anatomy at the level of repair in these women will be abnormal, including the ligamentum flavum and epidural space, rendering loss-of-resistance techniques impossible. Furthermore, posterior spinal cord tethering may be present, even in the context of prior detethering at the time of surgery, leaving it vulnerable to needle trauma.^36^ A posterior tethered cord with intact sensation is considered an absolute contraindication to neuraxial anaesthesia for this reason.^37^^,^^38^

Women with a history of spinal dysraphism should be seen well in advance of delivery in the preassessment clinic. The presence of a cerebral shunt and latex allergy should be determined and a full neurological assessment should be performed to ascertain a neurological level. Imaging, namely MRI, should be sought to allow identification of the level of termination of the spinal cord, the presence of tethering, assessment of CSF cistern volume and the presence of masses such as lipomata. MRI is also useful to identify normal anatomical levels where neuraxial anaesthesia may be possible.

**Fig 5:**
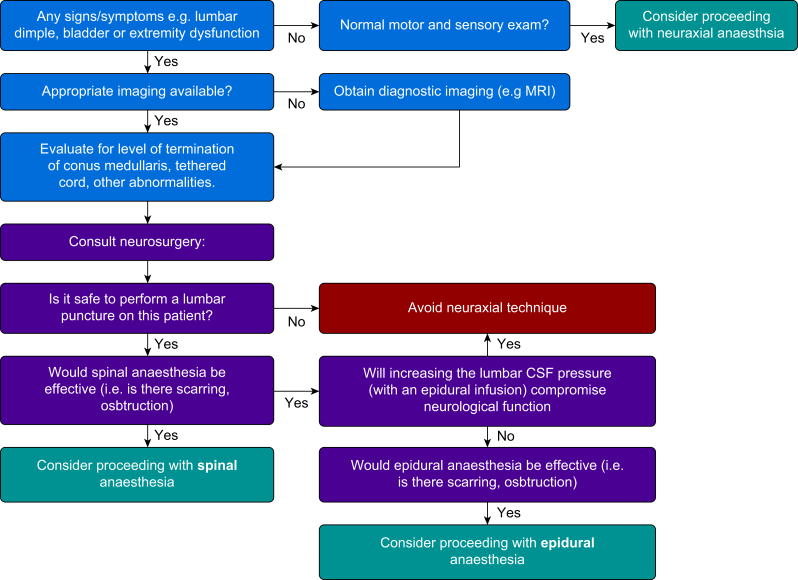
An algorithm to guide decision making around neuraxial anaesthesia in patients with spinal dysraphism. (Adapted from original work, courtesy of Dr Lisa Leffert.)

Based on the clinical and radiological investigations, options for labour analgesia and anaesthesia should be discussed with the patient. Women with a sensory level above T10 may not require labour analgesia, although anaesthesia above T4 is required for Caesarean delivery. Autonomic dysreflexia is rare in this population as the majority of lesions occur in the lumbosacral region. In those with a sensory level above T6, consideration should be given to provision of anaesthesia to avoid provoking autonomic dysreflexia. Neuraxial anaesthesia may be possible in certain cases. For women with isolated bony abnormalities without neural or meningeal involvement, it is reasonable to proceed with either epidural or intrathecal anaesthesia. There is a theoretical risk that the ligamentum flavum may be abnormal at the level of these defects, which may increase the risk of dural puncture, and as such some reports recommend avoiding this level.^39^ In women with previous repair, neuraxial anaesthesia above the level of the abnormality has been described.^37^ In these situations, MRI is helpful to locate a site where the epidural anatomy is normal, and the spinal cord is not tethered posteriorly. Ultrasound can then be used to locate the chosen level at the bedside. In terms of epidural anaesthesia, patients should be counselled that analgesia may be asymmetric or suboptimal because of abnormalities of the epidural space. Lower epidural bolus doses should be used in these individuals for the same reason. Successful spinal anaesthesia including both single shot, CSE and spinal catheter insertion at lumbar and low thoracic levels distant to the level of the lesion have also been described.^40^, ^41^, ^42^ An algorithm to guide decision making in patients with spinal dysraphism is provided in Figure 5.

## Management of the patient in whom a neuraxial technique cannot be performed

The decision to pursue or to avoid a neuraxial technique in a patient with structural spinal disease is nuanced, and often requires an assessment and balance of competing risks. Absolute structural contraindications to neuraxial anaesthesia are limited. A neuraxial approach may be considered in the presence of relative contraindications if the alternative poses more risk to the patient.

There is a wide variety of both pharmacological and non-pharmacological techniques for labour analgesia where a neuraxial approach is not possible. Remifentanil patient-controlled analgesia for labour is increasingly popular and has recently been reviewed in this journal.^43^ There is also some evidence to support the use of inhaled nitrous oxide and flurane derivatives, relaxation, water immersion, acupuncture and massage.^44^

## Managing postdural puncture headache in the patient with structural spinal abnormalities

Management of postdural puncture headache in patients with structural spinal abnormalities may be challenging where the epidural space has been difficult or even impossible to access. Image-guided epidural blood patch has been described in postspinal surgery patients with complex anatomy and may be an effective and safe option.^45^

## Summary

Disorders of the lower back and vertebral column are common in women of childbearing age. In the majority of these individuals, neuraxial techniques are possible. With timely preassessment and appropriate clinical history and investigations, an effective plan for labour analgesia can be made with counselling around risks and possible difficulties that may be encountered.

## Acknowledgements

The authors would like to thank Dr C. Bowens for the images in Figure 4.

## Declaration of interests

The authors declare that they have no conflicts of interest.

## MCQs

The associated MCQs (to support CME/CPD activity) will be accessible at www.bjaed.org/cme/home by subscribers to *BJA Education*.

## References

[1] Weiniger C.F., Einav S., Elchalal U. (2018). Concurrent medical conditions among pregnant women - ignore at their peril: report from an antenatal anesthesia clinic. Isr J Health Policy Res.

[2] Anim-Somuah M., Smyth R.M., Jones L. (2011). Epidural versus non-epidural or no analgesia in labour. Cochrane Database Syst Rev.

[3] Orlikowski C.E., Dickinson J.E., Paech M.J., McDonald S.J., Nathan E. (2006). Intrapartum analgesia and its association with post-partum back pain and headache in nulliparous women. Aust N Z J Obstet Gynaecol.

[4] Hebl J.R., Horlocker T.T., Kopp S.L., Schroeder D.R. (2010). Neuraxial blockade in patients with preexisting spinal stenosis, lumbar disk disease, or prior spine surgery: efficacy and neurologic complications. Anesth Analg.

[5] Pitkänen M.T., Aromaa U., Cozanitis D.A., Förster J.G. (2013). Serious complications associated with spinal and epidural anaesthesia in Finland from 2000 to 2009. Acta Anaesthesiol Scand.

[6] de Sèze M.P., Sztark F., JanvierG Joseph PA. (2007). Severe and long-lasting complications of the nerve root and spinal cord after central neuraxial blockade. Anesth Analg.

[7] Moen V., Dahlgren N., Irestedt L. (2004). Severe neurological complications after central neuraxial blockades in Sweden 1990-1999. Anesthesiology.

[8] Chow J., Chen K., Sen R., Stanford R., Lowe S. (2008). Cauda equina syndrome post-Caesarean section. Aust N Z J Obstet Gynaecol.

[9] Neal J.M., Barrington M.J., Brull R. (2015). The second ASRA Practice Advisory on neurologic complications associated with regional anesthesia and pain medicine: executive summary 2015. Reg Anesth Pain Med.

[10] Ko J.Y., Leffert L.R. (2009). Clinical implications of neuraxial anesthesia in the parturient with scoliosis. Anesth Analg.

[11] Chan E.W., Gannon S.R., Shannon C.N., Martus J.E., Mencio G.A., Bonfield C.M. (2017). The impact of curve severity on obstetric complications and regional anesthesia utilization in pregnant patients with adolescent idiopathic scoliosis: a preliminary analysis. Neurosurg Focus.

[12] Yakhup A., Okada H., Kawagoe I., Sumikura H. (2023). Anesthesia outcomes of pregnant women with spinal diseases: a single-center case-series study. JA Clin Rep.

[13] Bauchat J.R., McCarthy R.J., Koski T.R., Wong C.A. (2015). Labor analgesia consumption and time to neuraxial catheter placement in women with a history of surgical correction for scoliosis: a case-matched study. Anesth Analg.

[14] Lirk P., Moriggl B., Colvin J. (2004). The incidence of lumbar ligamentum flavum midline gaps. Anesth Analg.

[15] Vermani E., Mittal R., Weeks A. (2010). Pelvic girdle pain and low back pain in pregnancy: a review. Pain Pract.

[16] Casagrande D., Gugala Z., Clark S.M., Lindsey R.W. (2015). Low back pain and pelvic girdle pain in pregnancy. J Am Acad Orthop Surg.

[17] Brinjikji W., Luetmer P.H., Comstock B. (2015). Systematic literature review of imaging features of spinal degeneration in asymptomatic populations. AJNR Am J Neuroradiol.

[18] LaBan M.M., Perrin J.C., Latimer F.R. (1983). Pregnancy and the herniated lumbar disc. Arch Phys Med Rehabil.

[19] Saraste H. (1986). Spondylolysis and pregnancy--a risk analysis. Acta Obstet Gynecol Scand.

[20] Kalichman L., Kim D.H., Li L., Guermazi A., Berkin V., Hunter D.J. (2009). Spondylolysis and spondylolisthesis: prevalence and association with low back pain in the adult community-based population. Spine (Phila Pa 1976).

[21] Vanti C., Ferrari S., Guccione A.A., Pillastrini P. (2021). Lumbar spondylolisthesis: STATE of the art on assessment and conservative treatment. Arch Physiother.

[22] Kalichman L., Hunter D.J. (2008). Diagnosis and conservative management of degenerative lumbar spondylolisthesis. Eur Spine J.

[23] Hresko M.T. (2013). Clinical practice. Idiopathic scoliosis in adolescents. N Engl J Med.

[24] Huang J. (2010). Paramedian approach for neuroaxial anesthesia in parturients with scoliosis. Anesth Analg.

[25] Perlas A., Chaparro L.E., Chin K.J. (2016). Lumbar neuraxial ultrasound for spinal and epidural anesthesia: a systematic review and meta-analysis. Reg Anesth Pain Med.

[26] Collier C.B. (2014). Neuraxial anaesthesia in patients with scoliosis. Br J Anaesth.

[27] Lenke L.G., Betz R.R., Clements D. (2002). Curve prevalence of a new classification of operative adolescent idiopathic scoliosis: does classification correlate with treatment?. Spine (Phila Pa 1976).

[28] Grabala P., Helenius I., Buchowski J.M., Larson A.N., Shah S.A. (2019). Back pain and outcomes of pregnancy after instrumented spinal fusion for adolescent idiopathic scoliosis. World Neurosurg.

[29] Smith P.S., Wilson R.C., Robinson A.P., Lyons G.R. (2003). Regional blockade for delivery in women with scoliosis or previous spinal surgery. Int J Obstet Anesth.

[30] Grotle M., Småstuen M.C., Fjeld O. (2019). Lumbar spine surgery across 15 years: trends, complications and reoperations in a longitudinal observational study from Norway. BMJ Open.

[31] Agopian A.J., Canfield M.A., Olney R.S. (2012). Spina bifida subtypes and sub-phenotypes by maternal race/ethnicity in the National Birth Defects Prevention Study. Am J Med Genet A.

[32] Blatter B.M., Lafeber A.B., Peters P.W., Roeleveld N., Verbeek A.L., Gabreëls F.J. (1997). Heterogeneity of spina bifida. Teratology.

[33] Boone D., Parsons D., Lachmann S.M., Sherwood T. (1985). Spina bifida occulta: lesion or anomaly?. Clin Radiol.

[34] Mitchell L.E., Adzick N.S., Melchionne J., Pasquariello P.S., Sutton L.N., Whitehead A.S. (2004). Spina bifida. Lancet.

[35] Niggemann B., Buck D., Michael T., Haberl H., Wahn U. (2000). Latex allergy in spina bifida: at the turning point?. J Allergy Clin Immunol.

[36] Hertzler D.A., DePowell J.J., Stevenson C.B., Mangano F.T. (2010). Tethered cord syndrome: a review of the literature from embryology to adult presentation. Neurosurg Focus.

[37] Murphy C.J., Stanley E., Kavanagh E., Lenane P.E., McCaul C.L. (2015). Spinal dysraphisms in the parturient: implications for perioperative anaesthetic care and labour analgesia. Int J Obstet Anesth.

[38] Walsh E., Zhang Y., Madden H., Lehrich J., Leffert Leffert L. (2021). Pragmatic approach to neuraxial anesthesia in obstetric patients with disorders of the vertebral column, spinal cord and neuromuscular system. Reg Anesth Pain Med.

[39] McGrady E.M., Davis A.G. (1988). Spina bifida occulta and epidural anaesthesia. Anaesthesia.

[40] Broome I.J. (1989). Spinal anaesthesia for Caesarean section in a patient with spina bifida cystica. Anaesth Intensive Care.

[41] Nuyten F., Gielen M. (1990). Spinal catheter anaesthesia for Caesarean section in a patient with spina bifida. Anaesthesia.

[42] Kuczkowski K.M., Zuniga G. (2007). Labor analgesia for the parturient with spina bifida. Acta Anaesthesiol Scand.

[43] Ronel I., Weiniger C.F. (2019). Non-regional analgesia for labour: remifentanil in obstetrics. BJA Educ.

[44] Jones L., Othman M., Dowswell T. (2012). Pain management for women in labour: an overview of systematic reviews. Cochrane Database Syst Rev.

[45] Wong A.K., Rasouli M.R., Ng A., Wang D. (2019). Targeted epidural blood patches under fluoroscopic guidance for incidental durotomies related to spine surgeries: a case series. J Pain Res.

